# Treatment Predictability of Two Clear Aligner Systems: A Retrospective Assessment of Invisalign Versus Eon Aligner

**DOI:** 10.3390/dj14040240

**Published:** 2026-04-15

**Authors:** Raghad Abdullah Algarni, Saeed N. Asiri, Abdallah Al-Ani

**Affiliations:** 1 Department of Orthodontics, College of Dentistry, Riyadh Elm University, Riyadh 12734, Saudi Arabia; raghad.a.algarni2022@student.riyadh.edu.sa; 2 Department of Pediatric Dentistry, College of Dentistry, Prince Sattam Bin Abdulaziz University, Al-Kharj 16273, Saudi Arabia; 3 Division of Vascular and Interventional Radiology, Boston Children’s Hospital, Boston, MA 02115, USA; abdullah.al-ani@childrens.harvard.edu

**Keywords:** aligner, eon aligner, Invisalign, diagnostics, agreement

## Abstract

**Background/Objectives**: To compare the efficacy of two aligner systems (Invisalign and Eon Aligner) across multiple linear and angular movements. **Methods**: A total sample of 80 patient cases (n = 40 in each group) was recruited retrospectively. Per case, 3 digital models were retrieved in the form of stereolithography (STL) files. Predicted and achieved tooth movements were measured using the 3Shape Clear Aligner Studio. Initial models were aligned on the predicted and achieved models to create superimposition. Differences in measurement between pre-treatment, predicted, and post-treatment scans were measured. Agreement between the two, Invisalign and Eon, was measured using the interclass correlation coefficient (ICC). **Results**: Both Invisalign (ICC = 0.82; 95% CI 0.66, 0.9) and Eon Aligner (ICC = 0.75; 95% CI 0.53, 0.87) have shown good agreement when calculating the average differences between the achieved and predicted interpremolar width values. Similar results were found for both intercanine width values (Invisalign: ICC = 0.96; 95% CI = 0.93, 0.98 vs. Eon Aligner: ICC = 0.98; 95% CI = 0.97, 0.99). In Eon cases, good to excellent agreement between the achieved and predicted models was observed for lateral (ICC = 0.89; 95% CI = 0.79, 0.94) and central (ICC = 0.93; 95% CI = 0.87, 0.96) mesiodistal rotations. Conversely, Invisalign displayed moderate strength of agreement for the lateral (ICC = 0.68; 95% CI = 0.40, 0.83) and central (ICC = 0.70; 95% CI = 0.44, 0.84) mesiodistal readings. While both aligners demonstrated some level of predictive capacity towards horizontal movements, they were unreliable in predicting vertical movements. Differences in magnitude of change between initial and achieved values between Eon and Invisalign were noted only for certain teeth in the case of horizontal and vertical movements. **Conclusions**: Both clear aligner therapy systems were able to achieve satisfactory outcomes in terms of inter-premolar and intercanine width changes. Eon Aligner, on the other hand, outperformed Invisalign in terms of rotational accuracy and horizontal movement precision. Notably, both systems demonstrated poor predictability for vertical movements and suffer from significant systemic bias requiring over-correction.

## 1. Introduction

Since the introduction of Invisalign (Align Technology Inc. [Tempe, AZ, USA]) appliances in 1998, clear aligners have gradually gained immense popularity among doctors and orthodontic patients [1]. Compared to traditional fixed appliances, clear aligner therapy (CAT) is comfortable, removable, and has a low esthetic impact [2]. Moreover, the advent of 3D treatment planning enables professionals to pre-visualize treatment outcomes [3], which further motivates patients for its adoption. With the continuous development of materials and the addition of attachments, the CAT scope of treatment has expanded beyond mild malocclusions [4,5].

Invisalign is considered the most widely used and popular CAT system within the field of orthodontics. Due to marketing strategies, the prevalence of teenagers demanding CAT for esthetic treatment has been increasing [6]. While CAT is associated with less prevalence and severity of apical root resorption and better overall periodontal health compared to fixed appliances [7], these systems are often critiqued for their low accuracy. Particularly, between the predicted and achieved clinical outcomes [8,9,10].

Despite the plethora of literature pertaining to Invisalign technology, its clinical performance was inconsistent throughout [11,12]. Moreover, a recent systematic review demonstrated that the majority of conducted research is methodologically heterogenous, upon which no confident clinical recommendations could be made [13]. Since the expiration of the Align Technology Invisalign patent in October 2017, a number of clear aligner brands have been launched into the market [14]. These different CAT systems vary in material, design, and manufacturing techniques. Such systems include Angel Aligner (Angelalign Technology Inc. [Shanghai, China], Spark (Ormco Corporation [Brea, CA, USA]), and Eon Aligner (Eon Dental [Amman, Jordan])) [15,16].

All currently available CAT systems demonstrate variable efficacy [12]. This variability cannot be confidently attributed to differences in CAT designs or poor methodological characteristics. Therefore, the choice of clinicians regarding these systems is often anecdotal, relying on clinical expertise, opinions of other experts, and an extremely heterogeneous body of literature [17]. This study aims to compare the efficacy of two aligner systems (Invisalign and Eon Aligner) across multiple linear and angular movements [18] during the initial phase of treatment, before refinements.

## 2. Materials and Methods

### 2.1. Study Design and Participants

This study adopted a retrospective design and was conducted in the Department of Orthodontics at Riyadh Elm University between January 2023 and January 2025. The adopted inclusion criteria were as follows: (1) adult patients 18 years of age or older, (2) had malocclusion (Angle’s Class I, II, or III), (3) mild-to-moderate crowding (2–6 mm) or spacing (1–6 mm), (4) were prescribed a non-extraction-based treatment plan by a dental professional, and (5) completed two sets of aligner scans with adequate field view. Patients were excluded if they had skeletal crossbites, missing teeth, craniofacial syndromes or malformations (e.g., cleft lip), or dental/teeth deformities. Furthermore, patients with a history of posterior teeth restorations or planned second molar movement were also excluded.

### 2.2. Sample Size Estimation

Based on the findings from a previous study done in a similar field [14], a total sample of 80 (n = 40 in each group) was estimated to be the minimum required sample size for the study with an effect size of 0.65, power of the study at 0.8, and the level of significance at 0.05. Sample power was calculated using the GPower 3.1 sample power calculator (Kiel University, Kiel, Germany).

### 2.3. Protocol

Recruited patients were treated by two different clear aligner brands. Group A was treated by Invisalign, while group B was treated by Eon Aligner. Patients were sampled consecutively from the records of clinics across the Kingdom of Saudi Arabia based on the aforementioned inclusion criteria. Of the 40 cases treated by Eon Aligner, 25 were females, and 15 were males. The cases’ composition in terms of malocclusion was 8 spacing, 24 crowding, 6 crowding with Class II, 1 crowding with Class III, and 1 spacing with Class II. As for those treated by Invisalign, 23 were females and 17 were males; there were 22 cases of crowding, 7 of spacing, 7 of crowding with Class II, 1 of crowding with Class III, 2 of spacing with Class II, and 1 of spacing with Class III. The cases had minimal IPR planned, within a range of 0.2–0.3 mm per tooth, limited to the anterior region.

**Figure 1:**
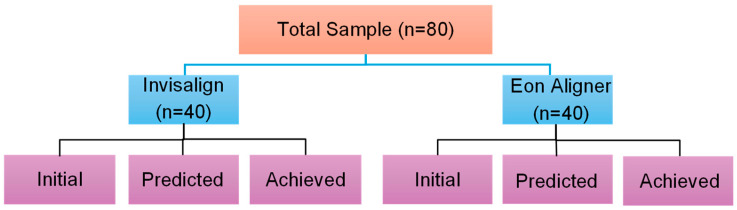
Study protocol. A total of 80 patient records were sampled, with 40 treated by the product of one of the two different brands. The initial, predicted, and achieved models were obtained in an STL format for each patient.

For each patient, 3 digital models were retrieved in the form of stereolithography (STL) files. The first model was the patient’s ‘initial’ scan prior to any treatment, the second model was the ‘predicted’ outcome taken from the last step of the treatment plan, and the third model was the ‘achieved’ result obtained from the patient’s pre-refinement phase scan, since this study aims to assess the efficacy of the first set of aligners without taking the refinement phase into consideration. The last ‘achieved’ model signified the actual outcome after the first stage of aligner wear [19]. Figure 1 demonstrates the study’s protocol.

**Figure 2:**
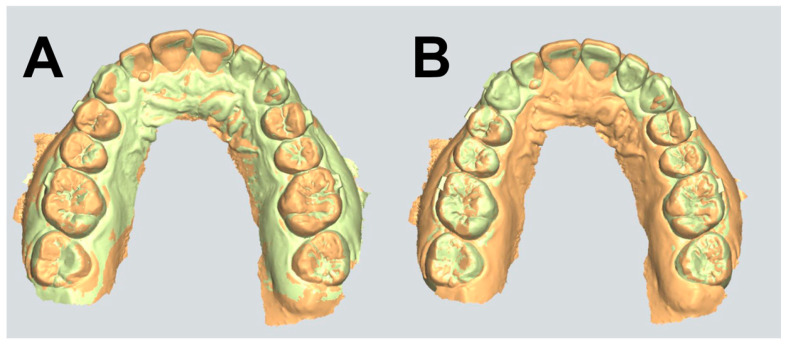
(**A**) The initial and predicted tooth models superimposed onto one another. (**B**) The initial and achieved tooth models superimposed onto one another.

For each patient, predicted and achieved tooth movements were measured using the 3Shape Clear Aligner Studio (3Shape, Copenhagen, Denmark). The software had demonstrated its accuracy, diagnostic reliability, and validity in digital orthodontic assessments [20]. The initial models were superimposed on the predicted models (refer to Figure 2) and then again similarly on the achieved ones. The resultant images allowed for the obtainment of tooth movement values by measuring the distance between the initial model and the predicted, and then the initial model and the achieved, thereby estimating a ‘predicted tooth movement’ and ‘achieved tooth movement’, respectively. This method was applied for horizontal, vertical, as well as mesiodistal rotations. Meanwhile, the intercanine and interpremolar widths were measured for both the upper and lower jaws of the 3 models.

### 2.4. Teeth Movement Measurement Protocol

The upper and lower jaws of both models (e.g., initial—predicted, initial—achieved) were aligned and superimposed together based on 3 corresponding points that remained stable and had no or minimal movement during orthodontic treatment. These included the mesiobuccal cusp tip of the right second molar, the center of the central fossae of the right second molar, and the mesiobuccal cusp tip of the left second molar.

**Figure 3:**
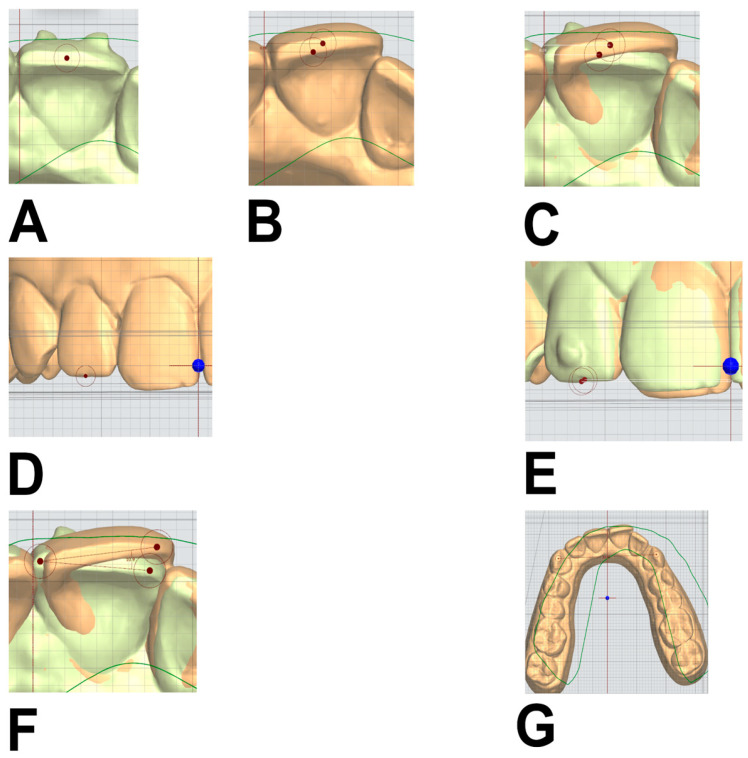
(**A**,**B**): Reference points marked on the centers of the incisal edges of the upper left central incisor on the initial and predicted models. (**C**): Measurement of the difference between the two reference points, corresponding to predicted horizontal movement. (**D**): Reference point marked on the center of the incisal edge of the upper right lateral incisor, frontally, on the initial model. (**E**): Second reference point, on the same landmark, marked on the predicted dental model, and the difference between the two calculated to obtain the predicted vertical movement. (**F**): Reference points marked on the incisal edge of the upper left central incisor on the initial and predicted models to calculate the predicted mesiodistal rotation. (**G**): Intercanine width of the upper jaw from the initial model, calculated by measuring the distance between the cusp tips of the canines.

For horizontal and vertical movements, distance was measured by measuring changes in a reference point marked on either the center of the incisal edge of anterior teeth or the buccal cusp tip of the canines and premolars. Horizontal movements were measured from an occlusal view, whereas vertical ones were measured from a frontal view (Figure 3A–E). Mesiodistal rotations were estimated by measuring the angle between two lines passing through the mesiodistal width of the same tooth on two different models (Figure 3F). Finally, the intercanine and interpremolar widths were measured as the distance between the cusp tips of the canines and the distance between the centers of the central fossae of the second premolars, respectively (Figure 3G). Measurements from both arches were included.

One examiner was calibrated through multiple measurements of the same model differences. The examiner completed 10 readings for all five measurements across two time points. T0 represented initial readings, while T1 represented the same readings after two weeks. The interclass correlation coefficient (ICC) values for all five measures ranged from 0.766 to 0.892. These values were considered satisfactory.

### 2.5. Statistical Analysis

All data was entered and cleaned using Microsoft Excel. The extracted data were presented in the form of descriptive statistics. For categorical variables, data were demonstrated as frequencies with their associated percentages. For continuous variables, data were demonstrated as means ± standard deviations. Normality of variables in isolation or as part of a group was determined through a combination of tests. These include the Shapiro–Wilk and Kolmogorov–Smirnov tests, histograms, and Q-Q plots. Such a rationale was adopted due to the extremely conservative nature of the earlier tests, which are sensitive to type II errors.

Agreement between predicted and achieved models was examined using ICC. The values of the ICC were interpreted as follows: poor agreement (0.0–0.5), moderate agreement (0.5–0.75), good agreement (0.75–0.9), and excellent agreement (0.9–1.0) [21]. Paired mean differences within either the Eon Aligner or Invisalign groups were explored using the Wilcoxon matched-pairs test. Similarly, intra-group mean differences were examined using the Welch’s *t*-test. Significant differences in means signify the presence of systematic bias between tools. A Bonferroni correction was applied to all reported *p*-values when the number of multiple tests exceeded 10. A *p*-value of less than 0.05 was considered statistically significant for all conducted tests. All analyses were pooled and conducted on SPSS version 23.0.

## 3. Results

Forty previously treated cases with available refinement scans from each of Eon Aligner and Invisalign were recruited in this study, adding up to a total of eighty cases. All of whom were characterized by mild to moderate malocclusions. Four cases were excluded from the study due to the absence of teeth. Table 1 demonstrates the ICC between the initial and predicted or achieved models. Table 2 demonstrates paired differences between predicted and achieved models. Table 3 demonstrated the mean differences between Eon Aligner and Invisalign.

### 3.1. Interpremolar Width

Both Invisalign and Eon aligners demonstrated good agreement between achieved and predicted interpremolar width outcomes. The differences between the two aligner brands were statistically non-significant, suggesting high predictability for this movement. Furthermore, significant differences between initial and achieved estimates confirm that both treatments were able to induce a meaningful transverse expansion. The magnitude of mean difference between achieved and predicted values was not significantly different between Invisalign and the Eon aligner.

### 3.2. Intercanine Width

In terms of intercanine width, both brands exhibited excellent agreement and strong reliability when comparing achieved and predicted values. Invisalign has shown non-significant differences between both predicted and achieved values as well as initial and achieved values, indicating that the planned change was insignificant in the first place. In contrast, the Eon aligner showed significant differences between both the predicted and achieved models. Despite these differences, there was no significant variation in the magnitude of the mean difference between the two treatment arms.

### 3.3. Mesiodistal Rotations

The Eon aligner demonstrated a better degree of agreement compared to Invisalign for mesiodistal rotations of both lateral and central incisors. However, both aligner brands demonstrated significant discrepancies between achieved and predicted values for the aforementioned rotations. Upon comparing the magnitude of the mean difference between predicted and achieved models, both brands showed no significant differences.

### 3.4. Horizontal and Vertical Movements

When comparing predicted to achieved movements, the Eon aligner showed good to excellent agreement for horizontal movement across various teeth, while Invisalign showed only moderate agreement. On the other hand, the predictability of both systems for vertical movements was poor. Invisalign was found to be unreliable, while the Eon aligner demonstrated moderate but fluctuating reliability, except for the first premolar teeth.

Differences between achieved and predicted values were statistically significant across both brands in terms of vertical and horizontal movements. One notable exception is the second premolar teeth treated by the Eon aligner. It appears that Invisalign exhibited significantly higher discrepancies between achieved and predicted values than the Eon aligner for the horizontal movement of the first premolar teeth and canines, as well as the vertical movements of both premolars.

## 4. Discussion

This investigation demonstrated excellent predictive reliability in terms of inter-premolar width measurements for both Invisalign and Eon Aligner, hence validating both CAT systems for prediction of transverse width changes. This finding aligns with Morales-Burruezo et al., who measured the effectiveness and predictability of the Invisalign system in the expansion of the arch and demonstrated high predictability for both inter-premolar and inter-canine width expansion [22]. Zhou et al. demonstrated that Invisalign’s greatest expansion efficacy was in the premolar area [23]. However, Lione et al. and Grunheid et al. noted that in non-extraction cases, maxillary arch expansion may not be fully attained [24,25].

Both Eon Aligner and Invisalign demonstrated significant differences between initial and achieved models in terms of inter-premolar width. Similarly, both CAT systems did not show significant differences between predicted and achieved models. These findings contrast those of Solano-Mendoza et al., who reported limitations in predicting width expansions for Invisalign, particularly within the posterior segments [26]. This discrepancy is probably owed to the growing technological advancement and evolution of aligner products, including Invisalign. Changes to production materials, gingival margin design innovations, and the addition of attachments, divots, and auxiliaries have been shown to improve the clinical outcomes associated with clear aligners [27]. Despite the presence of statistically significant differences between some measurements when comparing both brands, some of the mean differences may be considered small in magnitude. Camardella et al. recognize measurements greater than 0.4 mm in the transverse and sagittal planes as clinically relevant [28]. The differences in this study range between 0.06 and 0.34 mm, which fall within or close to accepted clinical tolerance limits.

In terms of inter-canine expansion, Invisalign did not achieve significant changes between initial and achieved width measurements. This finding is similarly observed across studies comparing CAT systems to traditional fixed orthodontic appliances, within which the latter did not display sufficient accuracy and effectiveness in terms of transverse arch expansion [29,30]. On the other hand, despite Eon Aligner’s high replicability, there was a significant difference between predicted and achieved values. This finding indicates that a systematic bias might be present in which the current software over/underestimates the magnitude of expansion. Interestingly, Caruso et al. suggest that over-estimation is inevitable and was observed for Invisalign, further promoting correction of digital predictions [31].

In the present study, Eon Aligner outperformed Invisalign in terms of predicting mesiodistal rotational movement across both lateral and central incisors. However, both CAT systems demonstrated significant differences between their achieved and predicted outcomes, thus indicating a systemic bias. This bias could originate from calibration errors, not accounting for random error, or simply regression to the mean. From a clinical perspective, these biases may be attributed to material insufficiency, biological resistance from teeth, unwanted movements, or poor compliance. These results confirm that CAT systems are limited in effectively rectifying rotational movement, irrespective of the system. These findings are consistent with the present literature [8,9,20,32,33]. Nonetheless, there exist reports that contradict such findings [3]. Despite the presence of bias, Eon Aligner demonstrated significant changes between the initial and achieved models. These could be attributed to differences in material stiffness, manufacturing design, and higher grip and force distribution around rotations [18]. Nonetheless, the overall findings from our study and those from the literature reaffirm the importance of individualized treatment within the context of case complexity and patient characteristics. However, the detected differences between incisor mesiodistal rotations (1.9–4.2°) may be considered clinically significant. This is because rotational inaccuracies in anterior teeth can compromise contact point alignment, result in black triangles, and increase the need for refinement stages.

Similarly, Eon Aligner outperformed Invisalign in terms of predictions; however, both CAT systems were in need of over-corrections. The disparity between predicted and achieved outcomes is well noted for Invisalign [22,25,34]. Kravitz et al. noted that clear aligners work best within linear translations [8]. However, increases in magnitude and complexity are associated with lower movement predictability. We believe that increased predictive efficacy of the Eon Aligner may lie in its better fit, which allows for smoother control over bodily tooth movements [18]. Yet, the need for over-corrections highlights the importance of meticulous treatment planning and supplementary modifications. This is particularly true for complex cases.

While both brands displayed a statistically significant difference between the achieved and predicted values for horizontal movements, some of those mean differences may be considered clinically irrelevant [28]. Invisalign’s mean differences, ranging between 0.206 and 0.341, are slightly greater than what was published by Charalampakis et al., who reported minor discrepancies, ranging between 0.20 and 0.25 mm [19]. Eon Aligner, on the other hand, exhibited lower ranges of mean differences, between 0.015 and 0.24, which is in agreement and even lower than those reported by Charalampakis et al.

On the other hand, both CAT systems demonstrated poor performance in terms of treating complex vertical adjustments, as demonstrated by their ability to predict vertical movements. These findings are in line with those of Krieger et al., which indicated that Invisalign is ineffective in controlling vertical movements, especially anterior intrusion [35]. The authors postulate that clear aligners could only achieve mild tipping and slight repositioning, lacking the capability to address significant vertical adjustments. Similarly, Drake et al. established that clear aligners were optimally adapted for linear displacements (e.g., space closure) but are unable to deal with vertical displacements due to insufficient force vectors and intrinsic design limitations, which prevent the application of consistent vertical forces necessary for precise movement [36]. Furthermore, Charalampakis et al. indicate that despite the technical advancements in CAT design and production, vertical adjustments with transparent aligners are unreliable without supplemental stabilization [19].

The discrepancies in predicting vertical movements could be attributed to three significant factors. Firstly, from a biomechanical perspective, clear aligners are limited by their reliance on ‘pushing forces’, which lack the vertical vector to achieve any movement within their respective plane [37]. Secondly, software prediction may predispose errors as they employ idealistic algorithms that may assume 1-to-1 transfer of force movements without accounting for stress relaxation or the complex ‘watermelon seed effect’ [38]. Finally, biological variability, in the form of bone density or periodontal ligament response variance, could introduce a standardized level of unpredictability that cannot be fully considered when designing treatment protocols [39].

Clinically, the study findings show that orthodontists should refrain from using clear aligners alone for complex vertical corrections. Instead, they should use auxiliaries such as attachments, elastics, over-correction, or hybrid orthodontic methods to achieve the best vertical movement outcomes. Further research must investigate material development and auxiliary mechanics to overcome these longstanding limitations [19,35,36]. Overall, it appears that precise movement depends upon the aligner retention, and this retention depends upon the attachment and type or quality of the material, rather than the thickness of the material [40].

### Limitations

Our findings should be interpreted within the context of the following limitations: The study utilized a retrospective approach, which may carry its own biases, weakening the causal inferences that can be made. Subsequent studies need to conduct randomized controlled trials to further assess the effectiveness of Eon Aligner in achieving planned orthodontic corrective movements. While the sample size of 40 patients per group is adequate, more extensive cohort studies with larger sample sizes are needed to improve the generalizability of these results. It should be noted that the outcome of treatment could have been affected by inherent differences in biological response, adherence to aligner wear protocol, the use of different attachment shapes and sizes, or anatomy. Such differences should be treated as confounders for future research and addressed through randomized recruitment.

Additionally, although 3Shape Orthoanalyzer software is widely accepted as a measurement tool in the digital sphere, imperfections that arise from digital superimposition may compromise the precision of measuring very small discrepancies. Moreover, we were unable to follow the gold standard of maxillary jaw superimposition through the palatal rugae due to their absence in the scans; we resorted to the use of second molars since they had no movement planned. However, it could be possible that unwanted movements did happen during treatment, which could have affected the accuracy of the superimposition, particularly between the initial and achieved models. Another limitation worth mentioning is that we combined data from the upper and lower jaws rather than presenting and interpreting them intra-arch; this point could be taken into consideration in future studies. Perhaps the most critical limitation is the absence of an evaluation of post-treatment stability within this study, jeopardizing the ability to assess the success of the orthodontic intervention in the long term. Consequently, it is essential for subsequent studies to utilize more rigorous designs in conjunction with long-term evaluation of stability to truly portray the efficacy of Invisalign as a treatment modality.

While the treatment of cases by multiple orthodontists may introduce bias, such an issue was mitigated through the implementation of a standardized treatment protocol, objective assessments using digital tools, and the calibration of observers.

## 5. Conclusions

This is the first study comparing the clinical efficacy of Eon Aligner aligners to Invisalign. We demonstrated that both CAT systems were able to achieve satisfactory outcomes in terms of inter-premolar and intercanine width changes. Eon Aligner, on the other hand, outperformed Invisalign in terms of rotational accuracy and horizontal movement precision. Notably, both systems demonstrated poor predictability for vertical movements, with Invisalign showing a particularly inconsistent ability to achieve planned vertical corrections. Nonetheless, both systems suffer from significant systemic bias requiring over-correction for most measured tooth movements, which should be further studied and traced.

## Figures and Tables

**Table 1:** Interclass correlation coefficients between predicted and achieved models.

		Invisalign				Eon Aligner			
		*p*-Value	ICC	95% CI Lower Bound	95% CI Upper Bound	*p*-Value	ICC	95% CI Lower Bound	95% CI Upper Bound
Premolar	IPW_Predicted_–IPW_Achieved_	<0.001	0.82	0.66	0.9	<0.001	0.75	0.53	0.87
Canines	ICW_Predicted_–ICW_Achieved_	<0.001	0.96	0.93	0.98	<0.001	0.98	0.97	0.99
Lateral Incisor	MD_Predicted_–MD_Achieved_	<0.001	0.68	0.4	0.83	<0.001	0.89	0.79	0.94
Central Incisor	MD_Predicted_–MD_Achieved_	<0.001	0.7	0.44	0.84	<0.001	0.93	0.87	0.96
2nd Premolar	H_Predicted_–H_Achieved_	<0.001	0.7	0.44	0.84	<0.001	0.82	0.66	0.9
1st Premolar	H_Predicted_–H_Achieved_	0.013	0.51	0.08	0.74	<0.001	0.98	0.96	0.99
Canine	H_Predicted_–H_Achieved_	<0.001	0.72	0.47	0.85	<0.001	0.91	0.83	0.95
Lateral incisor	H_Predicted_–H_Achieved_	<0.001	0.76	0.55	0.87	<0.001	0.88	0.78	0.94
Central incisor	H_Predicted_–H_Achieved_	<0.001	0.7	0.43	0.84	<0.001	0.95	0.91	0.97
2nd Premolar	V_Predicted_–V_Achieved_	0.07	0.38	−0.17	0.67	0.024	0.47	0.0032	0.72
1st Premolar	V_Predicted_–V_Achieved_	0.588	−0.074	−1.02	0.43	0.105	0.33	−0.257	0.65
Canine	V_Predicted_–V_Achieved_	0.347	0.118	−0.659	0.53	0.007	0.55	0.146	0.76
Lateral incisor	V_Predicted_–V_Achieved_	0.19	0.25	−0.419	0.6	<0.001	0.69	0.42	0.84
Central incisor	V_Predicted_–V_Achieved_	0.78	−0.28	−1.4	0.32	<0.001	0.62	0.29	0.8

IPW: Inter-premolar width, ICW: Intercanine width, MD: Mesiodistal rotations, H: Horizontal, V: Vertical.

**Table 2:** Mean differences between initial, predicted, and achieved models per aligner type.

		Invisalign				Eon Aligner			
		*p*-Value	MD	95% CI Lower Bound	95% CI Upper Bound	*p*-Value	MD	95% CI Lower Bound	95% CI Upper Bound
Premolar	IPW_Predicted_–IPW_Achieved_	0.7733	−0.113	−0.901	0.675	0.2148	0.557	−0.336	1.451
Premolar	IPW_Initial_–IPW_Achieved_	0.0224	−0.935	−1.732	−0.139	0.0335	−1.015	−1.947	−0.083
Canines	ICW_Predicted_–ICW_Achieved_	0.7637	0.03	−0.174	0.235	0.0196	0.1532	0.0258	0.2806
Canines	ICW_Initial_–ICW_Achieved_	0.8718	−0.034	−0.459	0.39	0.0378	−0.586	−1.137	−0.0345
Lateral Incisor	MD_Predicted_–MD_Achieved_	<0.001	4.237	2.112	3.361	<0.001	2.809	1.666	3.951
Central Incisor	MD_Predicted_–MD_Achieved_	<0.001	4.049	1.98	6.119	0.001	1.867	0.777	2.956
2nd Premolar	H_Predicted_–H_Achieved_	<0.001	0.227	0.119	0.335	0.8932	−0.015	−0.252	0.221
1st Premolar	H_Predicted_–H_Achieved_	<0.001	0.272	0.16	0.384	0.003	0.067	0.023	0.112
Canine	H_Predicted_–H_Achieved_	<0.001	0.25	0.154	0.346	0.0312	0.083	0.007	0.159
Lateral incisor	H_Predicted_–H_Achieved_	0.015	0.206	0.041	0.371	<0.001	0.24	0.14	0.341
Central incisor	H_Predicted_–H_Achieved_	0.0024	0.341	0.127	0.555	<0.001	0.183	0.09	0.276
2nd Premolar	V_Predicted_–V_Achieved_	0.033	0.067	0.005	0.129	0.1261	−0.059	−0.136	0.017
1st Premolar	V_Predicted_–V_Achieved_	<0.001	0.167	0.1	0.235	0.0426	−0.074	−0.164	−0.002
Canine	V_Predicted_–V_Achieved_	<0.001	0.257	0.143	0.371	0.001	0.13	0.0531	0.207
Lateral incisor	V_Predicted_–V_Achieved_	<0.001	0.254	0.123	0.384	<0.001	0.241	0.139	0.343
Central incisor	V_Predicted_–V_Achieved_	0.0017	0.284	0.113	0.454	0.003	0.218	0.106	0.329

IPW: Inter-premolar width, ICW: Intercanine width, MD: Mesiodistal rotations, H: Horizontal, V: Vertical.

**Table 3:** Mean differences between Eon and Invisalign in terms of magnitude of difference between predicted and achieved linear or angular movements.

		MD (Eon Aligner)	MD (Invisalign)	Difference	95% CI	*p*-Value
Premolar	IPW_Predicted_–IPW_Achieved_	0.56	−0.11	0.67	−0.50, 1.8	0.3
Canines	ICW_Predicted_–ICW_Achieved_	0.15	0.03	0.12	−0.12, 0.36	0.3
Lateral Incisor	MD_Predicted_–MD_Achieved_	2.81	4.24	−1.4	−3.8, 0.96	0.2
Central Incisor	MD_Predicted_–MD_Achieved_	1.87	4.05	−2.2	−4.5, 0.13	0.064
2nd Premolar	H_Predicted_–H_Achieved_	−0.02	0.23	−0.24	−0.50, 0.01	0.064
1st Premolar	H_Predicted_–H_Achieved_	0.07	0.27	−0.2	−0.32, −0.08	0.001
Canine	H_Predicted_–H_Achieved_	0.08	0.25	−0.17	−0.29, −0.05	0.007
Lateral incisor	H_Predicted_–H_Achieved_	0.24	0.21	0.03	−0.16, 0.23	0.7
Central incisor	H_Predicted_–H_Achieved_	0.18	0.34	−0.16	-0.39, 0.07	0.2
2nd Premolar	V_Predicted_–V_Achieved_	−0.06	0.07	−0.13	−0.22, −0.03	0.011
1st Premolar	V_Predicted_–V_Achieved_	−0.07	0.17	−0.24	−0.34, −0.15	<0.001
Canine	V_Predicted_–V_Achieved_	0.13	0.26	−0.13	−0.26, 0.01	0.066
Lateral incisor	V_Predicted_–V_Achieved_	0.24	0.25	−0.01	−0.18, 0.15	0.9
Central incisor	V_Predicted_–V_Achieved_	0.22	0.28	−0.07	−0.27, 0.14	0.5

IPW: Inter-premolar width, ICW: Intercanine width, MD: Mesiodistal rotations, H: Horizontal, V: Vertical.

## Data Availability

The datasets used and analyzed during the current study are available from the corresponding author on reasonable request.
